# Perceptions of newly-qualified nurses performing compulsory community service in KwaZulu-Natal

**DOI:** 10.4102/curationis.v38i1.1474

**Published:** 2015-07-08

**Authors:** Selverani Govender, Petra Brysiewicz, Busisiwe Bhengu

**Affiliations:** 1 Department of Nursing, Durban University of Technology, South Africa; 2 School of Nursing and Public Health, University of KwaZulu-Natal, South Africa

## Abstract

**Background:**

Compulsory community service (CCS) for nurses commenced in South Africa in January 2008 after it was legislated in the new *Nursing Act* (Act No. 33 of 2005). Nurses completing their registered nurse programme are registered as community nurse practitioners (CNPs) during the CCS period and make up the largest number of health professionals serving CCS. Whilst health institutions have welcomed CNPs as additional resources for the shortage of nursing staff, no structured guidelines have been provided at a regional level as to how these nurses should be utilised or managed during the CCS year. To date, no large-scale study has been conducted on nurses carrying out CCS in order to generalise the findings.

**Objectives:**

To establish the perceptions of newly-qualified nurses carrying out CCS in KwaZulu-Natal, South Africa.

**Method:**

A quantitative survey design was used to obtain data from a randomly selected sample of the 2012 cohort of nurses carrying out CCS in KwaZulu-Natal.

**Results:**

CNPs have a positive attitude toward CCS and perceive themselves as being well prepared for the year of community service in terms of knowledge, skills and ability to administer nursing care. They identified positive benefits of the year of community service. The concerns raised were limited orientation and support; and a few CNPs experienced problems of acceptance by the nurses with whom they work.

**Conclusion:**

It is recommended that all health institutions who receive CNPs develop structured orientation and support for these nurses in order to promote their development, thereby enhancing their benefit to the communities they serve.

## Introduction

One of the South African government's responses to the challenge of limited human resources in the health sector was the introduction of compulsory community service (CCS) for all health professionals registering their qualifications for the first time with the Health Professions Council of South Africa (HPCSA) in 1997 (Republic of South Africa 1997). Doctors commenced CCS in 1998 as legislated in the *Medical, Dental and Supplementary Health Service Professions Amendment Act*, No. 89 of 1997 (‘the Act’) (Republic of South Africa 1997), followed by other health professionals in subsequent years. The intention of the Act was to retain newly-qualified health professionals in the country immediately after they qualify. According to Hatcher *et al*. (2014:1–2), the Department of Health's (DOH) main objective was to promote ‘equitable distribution’ of health services to the people of South Africa and for health professionals to develop further practical skills, knowledge, critical thinking and professional behaviour during the period of CCS.

The former Minister of Health, Dr Manto Tshabalala-Msimang, motivated for newly-qualified nurses to be included in CCS in January 2005 (Ndaki 2004); and legislation followed in the form of the *Nursing Act*, No. 33 of 2005 (Republic of South Africa 2006). The implementation of CCS for nurses commenced in January 2008 and is regulated by the South African Nursing Council (SANC). Regulation 765 of 24 August 2007 has been included as a section in the *Nursing Act* (SANC 2007) for nurses who complete the four-year nursing diploma or degree for registration as a nurse (General Community and Psychiatry) and as a midwife to ‘practise a profession in a prescribed category’, to carry out one year of remunerated CCS (Republic of South Africa 2006). To accommodate CCS, the concept of ‘Community Nurse Practitioner’ (CNP) was introduced in South Africa. However, upon registration as a CNP, he/she is guided by the code of conduct of a professional nurse and midwife (SANC 2007).

### Literature review

Various studies have explored the CCS carried out by different categories of health professionals who register with the HPCSA. More recently, Hatcher *et al.* (2014) conducted a national cross-sectional study to determine the satisfaction levels of doctors and dentists who performed CCS. The study found that community service officers who expressed high satisfaction with CCS, also benefitted from professional development, suggesting the need for continued support of community service health professionals in underserved areas. Pillay and Harvey’s (2006) survey of the experiences of 52 clinical psychologists serving CCS for the first time found that more than 50% experienced role confusion and difficulties in communicating with patients because of language differences, even though they felt they made a difference to the communities they served and had gained confidence in their professional ability. Visser *et al.* (2006) examined the experiences and attitudes of dieticians carrying out CCS whilst Parker *et al.* (2013) found that even though dieticians were well prepared for CCS, aspects of their educational preparation required revision in order to ensure their readiness to serve the communities in which they were placed. Khan, Knight and Esterhuizen’s (2009) survey of all speech, hearing and language therapists found that less than 50% were willing to serve in rural institutions after completing CCS, citing differences in the level of support and supervision, a lack of infrastructural support and resources, as well as language barriers as reasons. All of these studies concluded that, whilst the CCS policy is a sound government initiative, there is room for improvement.

The limited studies on the experiences of nurses performing CCS in South Africa since 2008 have been entirely qualitative in nature, focusing on the experiences and support offered to university graduates during this period. Roziers, Kyriacos and Ramugondo (2014) explored Cape Town university graduates’ experiences before and after CCS and found that these nurses’ experience of the realities of practice made them fearful and anxious, whilst Thopola, Kgole and Mamogobo (2013) found that Limpopo graduates’ professional development was challenged by staff shortages and a lack of resources. Two studies were conducted in Gauteng: Du Plessis and Seekoe (2013) investigated university graduates’ experience of midwifery and the respondents identified the complexities of practice and ambiguous situations as challenges; Tsotetsi (2012) found that the newly-qualified nurses had difficulty in adapting to the work environment because of limited support, verbal abuse, role conflict and unexpected workloads. To the authors’ knowledge, no study has explored the perceptions of CNPs from colleges and universities in KZN. This article, which is part of a larger study analysing the implementation of the CCS policy for nurses in KZN, reports on a survey on the perceptions of randomly selected CNPs from all KZN health districts.

### Problem statement

CCS aims to consolidate newly-qualified nurses’ knowledge, skills, behaviour patterns and critical thinking whilst developing them professionally for their role as registered nurses. International studies have proposed support for newly-qualified nurses in the form of structured mentorship or preceptorship for integration into their new roles (Bjerknes & Bjørk 2012; Duchsher 2009; Dyess & Sherman 2009). There are no formal structures to integrate newly-qualified South African nurses into institutions once they are employed. Morolong and Chabeli (2005) found that, whilst newly-qualified nurses had the necessary knowledge, skills and values for nursing practice, they lacked critical thinking in applying the nursing process, indicating that the supervision they receive could be improved.

The introduction of the CNP initially generated confusion and anxiety amongst newly-qualified nurses as well as the registered nurses who received them. CNPs were allocated to health units either as ‘finalist students’ awaiting registration, or as nurses with all the responsibilities of registered nurses. Inconsistencies in how CNPs are used or managed gave rise to the need to investigate how they perceive their year of CCS in order to make recommendations for future implementation of the CCS policy.

#### Research purpose

The purpose of this study was to explore the perceptions of nurses carrying out CCS in KZN.

#### Research objectives, questions and assumptions

The research objectives were to:

- describe the CNPs’ perceptions of the CCS policy
- establish the CNPs’ perceived preparedness for their role during CCS
- describe the perceived support received during the transition from student to CNP.

The research questions were:

- What were the CNPs’ perceived perceptions of positive and negative experiences during CCS?
- How prepared did the CNPs perceive themselves to be for their role during CCS?
- What perceived support did the CNPs receive during the transition from student to CNP?

The study was based on the assumptions that CNPs will perceive the CCS policy positively and receive adequate support during their transition from student to CNP; and that CNPs will be prepared adequately for their role during CCS.

#### Definition of key concepts

CCS is a one-year period of compulsory service rendered by nurses who have completed the training requirements for a four-year degree or diploma in nursing before registration as a nurse (general, community and psychiatry) and a midwife (SANC 2007).

CNP is a nurse who has completed the R. 425 programme for registration as a nurse (general, community and psychiatry) and a midwife who is carrying out CCS as legislated by regulation R. 765 (SANC 2007).

## Research design

The larger study was guided by the consolidated framework for implementation research (CFIR) designed by Damschroder *et al.* (2009). The CFIR framework consists of five domains, namely: the interventions, inner and outer settings, individuals involved and the implementation process (Damschroder *et al.* 2009). The CNPs in this article were a subset of participants from one of the individuals’ domains.

### Research approach and method

A cross-sectional descriptive survey design was used to gather quantitative data on CNPs’ perceptions of CCS.

#### Population, sampling and sample size

The study population comprised all the CNPs who commenced CCS in January 2012 at health institutions in KZN. The inclusion criterion was CNPs who had at least six months’ working experience. A list of the January 2012 CNPs allocated to health institutions in KZN was obtained from the KZN DOH – a total of 366 CNPs were placed in KZN health institutions from each of the three nursing education institutions (NEIs) that offer the four-year diploma or degree in nursing. Thirty per cent (*n* = 110) were selected at random from the list of CNPs (*N* = 366) who had completed at least six months of CCS to obtain a broad description of CNPs’ perceptions, until the sample size was reached. The sample size was determined by the institutions’ health sciences statistician who advised that a 30% sample size would provide a broad description of the perceptions of the CNPs. All CNPs on the allocation list were given a number from 1 to 366 and the numbers were placed in a large bowl. The required sample of 110 was drawn using the fish bowl technique. An additional five CNPs were drawn randomly to allow for attrition (Polit & Beck 2008).

#### Context of study

The study took place in all the health districts in KZN, namely, Amajuba, eThekwini, UMgungundlovu, UMzinyathi, Umkhanyakude, Zululand, Sisonke, UGu, UThukela, ILembe and UThungulu. The latter eight districts are considered rural districts. Institutions in urban districts located close to a rural area were considered to be semi-urban.

#### Research instrument

The survey questionnaire was developed by the researcher from the literature obtained regarding community service for doctors (Reid 2001) and dieticians (Visser *et al.* 2006), as well as adapting the Casey Fink questionnaire that was used for the graduate nurse experience survey (Casey *et al.* 2004). Section A was structured to obtain demographic data, whilst section B contained questions on a four-point Likert scale from 4 to 1 for ‘strongly agree’, ‘agree’, ‘disagree’ and ‘disagree strongly’, respectively, to solicit information on CNPs’ perceptions of CCS, their educational preparation, as well as their perceived experiences and perceptions whilst working as CNPs. Section C presented multiple response items on perceived difficulties or satisfaction as well as an open-ended question for additional comments on CCS.

#### Data collection

The survey questionnaire was administered in person by the researcher who visited the different health institutions where the CNPs were placed, from September to December 2012. After consultation with the unit managers, the consent forms were signed by participants after being assured that their participation was voluntary. The CNPs were permitted to complete the questionnaires whilst on duty and the completed questionnaires were collected before leaving the health institution. If a participant was not present, the questionnaire was left with the relevant unit manager for the CNP to complete and return by means of a stamped and self-addressed envelope. Follow up was made on unreturned questionnaires by telephoning the institution or by a return visit.

#### Data treatment

The data obtained were coded and captured into IBM SPSS Statistics for Windows version 21.0 (IBM Corp., Armonk, NY 2012) before analysis to obtain descriptive statistics such as frequencies. The frequencies obtained from analysis of Section B were combined from the four categories into two, namely, strongly agree/agree and disagree/strongly disagree for presentation of results and discussion. The comments made in response to the open-ended question in section C were read and re-read so as to determine common codes which were then categorised according to the different sections in the questionnaire.

## Ethical considerations

Ethical approval to conduct the study was obtained from the University of KwaZulu-Natal (UKZN) (reference number HSS/1245/011D) and KZN DOH Ethics Committees. Permission to collect the data was obtained from the gatekeepers. The KZN provincial DOH and the health district managers were approached to obtain permission from the various health institutions where the CNPs were allocated. Participant information sheets were provided to the survey participants and they were told that participation in the study was voluntary. The ethical principles of respect, autonomy and confidentiality were observed. Minimal risks were attached to participation and participants were informed that they were free to withdraw from the study at any time. Written informed consent was obtained from participants before they completed the questionnaires. Confidentiality was assured by using numbers rather than names on the questionnaires.

## Reliability and validity

Random selection of participants avoided threats to external validity whilst internal validity was ensured by review of the survey questionnaire by academic experts before being pre-tested on a convenience sample of five CNPs from a health institution situated close to the researcher. After reviewing the responses from the pre-test with nursing academic experts it was concluded that the instrument achieved content validity (the actual results of the pre-test were not included in the study). Slight changes were made to the instrument for easier completion and coding.

The questions in Section B were assessed for internal consistency through Cronbach's alpha (α) for reliability. The reliability coefficient was 0.725 for Questions 1–8 and 0.759 for Questions 9–23, whilst Section C scored 0.822 and the average score obtained was 0.768, indicating that the instrument had acceptable internal consistency (Polit & Beck 2008).

## Results

A response rate of 107 (93%) was obtained from the 115 questionnaires administered. Eighty-eight CNPs commented in the open-ended question on CCS for nurses. The findings from the comments were grouped for relevance to the CCS’ perceptions and experiences.

### Demographic data

Most of the CNPs, 71% (*n* = 76) fell into the 21 to 27 years age group whilst the remaining 31 participants (28.9%) were between the ages of 28 and 33 years. There were more women (76.6%; *n* = 82) than men (23.4%; *n* = 25). The majority of the participants (79.45%; *n* = 85) attended the regional College of Nursing and qualified with the four-year diploma in nursing; 83.2% (*n* = 89) were allocated to their first choice of health institution, whilst only a small percentage (6.5%; *n* = 7) were placed in areas which were not amongst their choices. A small percentage (28.6%; *n* = 30) received ongoing orientation, whilst 59 % (*n* = 62) of the participants received less than one week's orientation (see Table 1).

**TABLE 1:** Demographics of community nurse practitioners.

Variables	*n*	%
**Gender of CNPs (*n* = 107)**		
Male	25	23.4
Female	82	76.6
**Nursing school attended by participants (*n* = 107)**		
College of Nursing	85	79.4
University	22	20.5
**Location of health institution where placed (*n* = 107)**		
Rural	53	49.5
Urban	29	27.1
Semi-urban	25	23.4
**Previous work experience as a nurse (*n* = 104)**		
Yes	27	26
No	77	74
**Allocation versus choice of institution (*n* = 107)**		
1st choice	89	83.2
2nd choice	3	2.8
3rd choice	5	4.7
4th choice	1	0.9
5th choice	2	1.9
No choice	7	6.5
**Length of orientation given (*n* = 105)**		
Still ongoing	30	28.6
Less than one week	62	59.0
2 weeks	9	8.5
4 weeks	1	1.0
More than 4 weeks	2	1.9
None	1	1.0

### Perceptions of the compulsory community service policy

Eighty-nine (83.2%) of the participants agreed that the CCS policy is a good one and 86% (*n =* 92) agreed that they understood the objectives of this policy (see Table 2). Eighty-six percent (*n =* 92) of the participants agreed that they perceived satisfaction with their allocated clinical site; this is congruent with the participants’ choice of placement in section A. This comment supports the CNPs’ agreement:

‘The CSN [*CNP*] programme is good because it exposes us and prepares us to be able to stand on our own. We have developed a lot of skills in the units we have been allocated in. One year is sufficient to learn in the hospital you are placed in.’ (P18, female, 28 years)

**TABLE 2:** Community nurse practitioners’ perceptions of compulsory community service (*n* = 107).

Variables	Frequency (%) Strongly Agree/Agree	Frequency (%) Disagree/Strongly Disagree	*N*
**Perceptions about the CCS policy**			
The compulsory community service is a good policy to retain nurses in needed areas	89 (83.2)	18 (16.8)	107
I understand the objectives of the CCS policy for nurses	92 (86)	15 (14)	107
I am satisfied with my allocated clinical site*	89 (86.9)	13 (12.2)	102
**Perceptions about ability to communicate and take charge of situations**			
My nursing education has prepared me adequately to carry out my professional role*	102 (95.4)	4 (3.7)	106
I am able to carry out skills I acquired during my training	107 (100)	0 (0)	107
I feel confident when communicating with doctors about patients conditions	105 (98.1)	2 (1.9)	107
I am comfortable to delegate tasks to students and other staff under me	97 (90.6)	10 (9.4)	107
**Perceptions about confidence with patient care activities as a CNP**			
I have difficulty in prioritising patient care needs	13 (12.2)	94 (87.9)	107
I am overwhelmed by patient care responsibilities and workload in the unit	54 (50.4)	53 (49.5)	107
I feel comfortable making care decisions for my patients	104 (97.2)	3 (2.8)	107
I feel the job expectations of me are realistic as a CNP	87 (81.3)	20 (19.8)	107
I feel that I may harm a patient due to lack of knowledge and experience	11 (10.2)	96 (89.7)	107
**Perceptions on the support received during CCS**			
I am supported by nurses in the unit	103 (96.3)	4 (3.7)	107
My mentor/ preceptor is supportive towards my adjustment in the health care institution	66 (61.7)	41 (38.3)	107
I am at ease in asking for help from the mentor/ registered nurse in the unit	103 (96.3)	4 (3.7)	107
There are positive role models for me to observe in my unit*	95 (88.8)	11 (11.2)	106
I was given orientation by the healthcare institution when I arrived to do my CCS*	81 (75.7)	25 (24.3)	106
The orientation program that I received was useful*	79 (74.5)	27 (25.5)	106
I have a mentor to guide me in developing confidence*	68 (64.2)	38 (35.8)	106
My unit manager gives me encouragement and feedback about my work performance	78 (72.9)	29 (27.1)	107
**Perceptions of CNPs on their personal satisfaction during CCS**			
I feel my work is exciting and challenging	99 (92.5)	8 (7.5)	107
I am supported by my friends/ family while carrying out my CCS	103 (96.3)	4 (3.7)	107

CCS, compulsory community service; CNP, community nurse practitioner.

*, not all participants responded.

### Community nurse practitioners**’** preparedness for their nursing role

The overwhelming majority of the participants (95.4%; *n =* 102) felt that they were sufficiently prepared for their nursing role and 100% (*n =* 107) were able to offer care with confidence. Only 12.2% (*n =* 13) agreed that they had difficulty in prioritising patients’ care needs and 10.2% (*n* = 11) felt that they might harm a patient because of a lack of knowledge. Just over half of the participants (50.4%; *n =* 54) agreed that they were overwhelmed by patient care (see Table 2). For example, a participant stated that:

‘The one challenge that I have as a CSN [*community service nurse*] is that sometimes I’m treated as a student and so duties that are not under my scope even if there is someone else who can do it [*who is in that specific category*]. Although I am still learning other things, but I still feel undervalued. I’m only recognised when there is a shortage.’ (P33, female, 24 years).

### Support received during the transition from student to community nurse practitioner

The majority of the participants (96.3%; *n =* 103) agreed that they were supported by nurses in the unit and 61.7% (*n =* 66) agreed that their mentors and/or preceptors were supportive in helping them adjust to the healthcare institution (see Table 2). There were 68 participants (64.2%) who agreed that their mentors guided them in developing confidence; 72.9% (*n =* 78) agreed that they received encouragement and feedback from the unit manager and most participants (96.3%; *n =* 103), agreed that they felt at ease when seeking help from the registered nurse in the ward. However, in response to the open-ended question, a few CNPs (8.4%; *n* = 9) stated that they would have benefited from mentoring before being handed huge responsibilities:

‘Nobody is supervising [*us*] because we are almost of the same level. Yet the drainage [*catchment*] area is big and lots of patient are seen in one day and get admitted with.’ (P8, male, 31 years)

### Factors that could have affected the transition from student to community nurse practitioner

The two main difficulties experienced by the CNPs were workload and role expectations (see Table 3). In terms of role expectations, most participants (60.7%; *n* = 65), agreed that the difficulties varied from autonomy, to more responsibility, having to be a preceptor to students and being left in charge of the unit. One CNP commented:

‘In some other departments com serve [*CNPs*] are overworked and staff does not recognise them as real professional nurse[*s*]. So I think people has [*have*] to be educated about com serve.’ (P59, female, 26 years)

**TABLE 3:** Factors affecting transitioning from student to community nurse practitioner (*n* = 107).

Variables	Frequency (%) Agree	Frequency (%) Disagree	*N*
**Perceived difficulties during transitioning**			
Role expectations	65 (60.7)	42 (39.3)	107
Lack of confidence	26 (24.3)	81 (75.7)	107
Workload	66 (61.7)	41 (38.3)	107
Orientation issues	41 (38.3)	66 (61.7)	107
**Aspects that could have made the CNP feel more supported into the unit/ health institution**			
Improved orientation	61 (57.0)	46 (43.0)	107
Increased support	54 (50.5)	53 (49.5)	107
Unit socialisation	35 (32.7)	72 (67.3)	107
Improved work environment	67 (62.6)	40 (37.4)	107

CNP, community nurse practitioner.

Sixty-six (61.7%) participants indicated difficulties with their workload, including organising, prioritising, being overwhelmed, patient and/or staff ratios and patient acuity. Another participant said:

‘I feel that CSN [*CNP*] should be gradually introduced to work system and be given feedback regularly so they can understand their shortcomings and be able to improve without feeling overwhelmed. I believe mentor could play a huge role in guarding [*guiding*] and encouraging them.’ (P74, female, 33 years)

Orientation issues were also identified as problematic by 38.3% (*n* = 41) of the participants whilst 57% (*n* = 61) agreed that improved orientation would have made them feel more supported in the unit or health institution (see Table 3). Fifty-four (50.5%) of the participants agreed that increased support would have been beneficial. One CNP commented that:

‘I was not happy to be put in charge of the unit on night duty during my first month of community service. At least a community service nurse could get a one week orientation before starting the job I would be happy and not to be on night duty for the first six months of community services [*service*].’ (P4, female, 31 years)

Orientation supports nurses in making the transition to their new role. Sixty-seven (62.6%) participants agreed that an improved work environment would also have assisted in this transition.

### Most satisfying and least satisfying experiences

Approximately 56% (*n* = 60) participants (see Table 4) were satisfied with their work during CCS and 72% (*n* = 77) felt supported by their peers. Working with patients and their families was satisfying for 57.9% (*n* = 62) of the participants. Many CNPs also stated that they gained from being exposed to different institutional settings. Half of the participants (50.5%; *n* = 54) agreed that outdated facilities and equipment and cramped workspaces were the least satisfying aspects of their work experience, whilst 49.5% (*n* = 53) disagreed with this statement.

**TABLE 4:** Most satisfying and least satisfying aspects of work environment (*n* = 107).

Variables	Frequency (%) Agree	Frequency (%) Disagree	*N*
**Aspects of the CNPs work environment that were most satisfying**			
Peer support	77 (72.0)	30 (28.0)	107
Patients and families	62 (57.9)	45 (42.1)	107
CNP role	60 (56.1)	47 (38.3)	107
Positive work environment	41 (38.3)	66 (61.7)	107
**Aspects of the CNPs work environment that were least satisfying**			
Nursing work environment	51 (47.7)	56 (52.3)	107
Outdated systems	54 (50.5)	53 (49.5)	107
Interpersonal relationships	47 (43.9)	60 (56.1)	107
Orientation	48 (44.9)	58 (54.2)	106

CNP, community nurse practitioner.

One of the CNPs experienced being left out of discussions in departments:

‘Sometime you find being left out in some discussions in the department due to you [*sic*] not being stationed in that unit. Secondly for staff upgrading … because you are not [*a*] permanent worker in the institution you found [*find* *yourself*] being left out when sending staff for workshop, [*and*] symposium.’ (P43, female, 45 years)

A few CNPs (9.3%; *n* = 10) felt that they had no clear role. They had completed their student training but were sometimes not recognised by the registered nurse(s):

‘Nurses do not recognise you as a professional nurse like other professional nurses, because of this specific name ‘community service nurse’ managers are making this community service nurse as a profession, not as a service that you are rendering; but also you are expected to act as a professional nurse with experience when they are short staffed of professional nurses.’ (P42, female, 29 years)

The majority of participants agreed that their role as CNPs (56.1%; *n =* 60), the patients and families (57.9%; *n =* 62) and support they received from peers (70%; *n =* 77) led to high levels of personal satisfaction.

## Discussion

The demographic data indicated that most CNPs fell within the 21 to 27 year age group whilst a small percentage of CNPs fell between the ages of 28 to 33 years. These age ranges may be considered acceptable considering the average age of graduates in Caliskan and Ergun’s (2012) study was 24.05 years. It was also below the average age of those that were reported to be studying for the degree in nursing in Western Cape (Jeggels, Traut & Africa 2013). The greater number of women (76.6%; *n* = 82) suggests a dominance of women for nursing, as confirmed by Dyess and Sherman (2009) and by Caliskan and Ergun (2012) in their study on graduate nurses.

Most participants attended the College of Nursing for a diploma in nursing as compared to the fewer numbers that attended university to pursue the degree in nursing; this could be attributed to more nursing student posts being available in the provincial College of Nursing. The greater proportion of CNPs were allocated to their first choice of health institution, suggesting that this aspect of the CCS policy was agreeable to the majority of the participants and is similar to results in Hatcher *et al.*’s (2014) study. The results also were true for the assumption that CNPs will perceive the CCS policy as positive.

Only a small number of participants received ongoing orientation, whilst the majority received less than one week's orientation. Orientation programmes are helpful with regard to helping newly-qualified nurses to transition into their professional role and can decrease the reality shock when nurses commence working (Caliskan & Ergun 2012). Lack of sufficient orientation was also revealed as one of the contributory factors for a lack of confidence affecting newly-qualified nurses (Thopola *et al*. 2013). In addition, orientation is necessary for positive socialisation into the profession (Duteau 2012).

All of the participants felt sufficiently prepared for their nursing role and in their ability to offer care with confidence, supporting the assumption that CNPs were adequately prepared for their role during the CCS year. Doody, Tuohy and Deasy (2012) agreed that adequate preparation promotes smooth transition to the world of work, whilst feeling unprepared for the role of professional nurse can lead to stress, requiring additional support. Almost half of the CNPs in this study felt overwhelmed by patient care responsibilities, whilst a few commented on the need for support when commencing CCS. In Du Plessis and Seekoe’s (2013) study, newly-qualified nurses working as midwives were often challenged with difficult situations, but still managed to grow and develop as professionals during the CCS year.

The majority of participants in this study felt supported by nurses and mentors in the units who had helped them develop confidence by giving feedback and encouragement. They also felt at ease when seeking help from the registered nurse in the nursing unit, confirming the assumption that CNPs will receive adequate support during their transition from student to CNP. Johnstone, Kanitsaki and Currie (2008) proposed support for the first four weeks of work as well as at every rotation in order for the graduate nurse to understand the organisational processes. Long-term support during the transition from student to professional develops clinical judgment and enhances skills development (Dyess & Sherman 2009). Rush *et al*. (2013) recommended that those who mentor new graduates should be trained to do so, whilst Spector and Echternacht (2010) proposed that a model for transition to practice should be considered by institutions employing newly-qualified nurses at any level and that preceptors and new graduates should work together over a six-month transition period.

Despite the CNPs being subjected to stressful periods at the beginning of the CCS year, the majority were satisfied with their work experience and found working with patients and their families to be satisfying. Du Plessis and Seekoe’s (2013) study found that nurses also experienced job satisfaction when they were appreciated and consulted by colleagues. Satisfaction with support and supervision was also rated highly by doctors and dentists in Hatcher *et al*.’s (2014) study. Halfer and Graf (2006) noted that satisfaction during the transition year for new graduates was a positive factor that could be related to the period of adjustment whilst Casey *et al.* (2004) found that new nurses take six to 12 months to adjust to their new role. Experiencing satisfaction during CCS could improve the retention of nurses, which is one of its objectives (Hatcher *et al*. 2014).

Although CNPs felt confident in carrying out patient care activities; many felt overwhelmed by the responsibilities of patient care. The role expectations and workload of newly- qualified nurses have been highlighted by Duchsher (2009) as reasons for early resignations. Thopola *et al*.’s (2013) study in Gauteng found that CNPs engaged in CCS carried heavy workloads, with limited orientation and support. The workload difficulties experienced by the participants were similar to what Kramer describes as reality shock (Kramer 1974, as cited in Halfer & Graf 2006), which new workers experience in spite of spending many years preparing for the work they would eventually perform.

The negative experiences of the participants can be related to Dyess and Sherman’s (2009) study where nurses reported numerous instances of feeling utterly alone, influencing feelings of professional isolation. Confusion regarding CNPs’ role was one of the areas of concern noted by Tsotetsi (2012). As specified in Regulation 765 of 15 February 1985 (SANC 2007), the CNP follows the scope of practice of a professional nurse; until clearly specified for this category, role confusion will continue to be a dilemma for CNPs.

## Limitations of this study

This study only reports on the perceptions of CNPs in the KZN province and can therefore only be generalised to CNPs in this province.

## Recommendations

The following recommendations emanate from the results:

- Guidelines for orientation of the CNP at the beginning of CCS need to be standardised to enable the adjustment from student to CNP and to ensure other nurses are aware of what is expected from the CNPs.
- A job description should be developed for CNPs; this should differ from that of registered nurses and should be made available to all staff.
- A structured mentorship programme should be established to help guide them during their CCS.

## Conclusion

CCS has been well accepted by the CNPs that participated in this study; these results could be generalised to CNPs in KZN. The objectives of the CCS year to consolidate the skills and competence of professionals appear to be partially achieved. This is attributed to the varying levels of support offered to CNPs. The objective of providing resources to underserved areas also appears to have been achieved as 50% of the participants were allocated to rural areas. The participants’ negative experiences should, however, be taken into consideration when supporting CNPs.
